# The Role of Sulopenem in the Treatment of Uncomplicated Urinary Tract Infections: A Systematic Review and Meta-Analysis

**DOI:** 10.7759/cureus.87052

**Published:** 2025-06-30

**Authors:** Quang D La, Han B La, Francis Pryor, Noor Rajpoot, Noman Sadiq

**Affiliations:** 1 Medicine, The Innovative STEMagazine, College Station, USA; 2 Medicine, Texas A&M College of Medicine, Bryan, USA; 3 Medicine, Lake Erie College of Osteopathic Medicine, Erie, USA; 4 Medicine, Teaching Hospital Turbat, Turbat, PAK; 5 Physiology, Mekran Medical College, Turbat, PAK

**Keywords:** bacterial infection, beta-lactam antibiotics, sulopenem, uncomplicated urinary tract infection (uuti), uti, uuti

## Abstract

Uncomplicated urinary tract infections (uUTIs) rank as one of the most frequent bacterial infections, particularly in females, and antimicrobial resistance is complicating the situation more and more. So, first-line agents such as nitrofurantoin and trimethoprim-sulfamethoxazole are losing their beneficial effects. There is an urgent call for new therapies due to the very alarming global rise of extended-spectrum β-lactamase-producing bacterial isolates.

Sulopenem is a new antibiotic of the penem series available for both intravenous and oral administration, a potential candidate against multidrug-resistant Gram-negative organisms. It offers anti-β-lactamase stability, oral activity, and possible hospitalization duration reduction, hence, making it the best option for consideration.

This article conducts a review of the evidence surrounding sulopenem regarding efficacy, safety, and resistance mechanisms. According to Preferred Reporting Items for Systematic Reviews and Meta-Analyses (PRISMA) guidelines, clinical trials were searched in databases, including PubMed, Scopus, Embase, Medline, and the Cochrane Library. Five studies were selected that include randomized trials, comparative effectiveness research, and pharmacokinetic/pharmacodynamic (PK/PD) modeling studies. Main outcomes were microbiological cure, resolution of symptoms, recurrence of clinical symptoms, and adverse events.

Sulopenem was either non-inferior or superior to comparators in important subgroups. Ciprofloxacin-resistant infections showed better test-of-cure results with sulopenem, 62.6% versus 35.0%. Sulopenem/probenecid was equal or superior to amoxicillin/clavulanate, including among resistant strains. PK/PD modeling confirmed bactericidal concentrations for a sustained duration.

## Introduction and background

Urinary tract infections (UTIs) are among the most frequent bacterial infections worldwide, with uncomplicated UTIs (uUTIs) accounting for most infections, particularly in women [1]. Infections, typically from Escherichia coli and other Enterobacteriaceae members, account for exorbitant healthcare expenditure due to recurrent infections and rising antibiotic resistance [2]. First-line treatments, such as nitrofurantoin and trimethoprim-sulfamethoxazole, have seen their effectiveness decline in much of the world, leading to the deployment of alternative therapeutic compounds [3]. The emergence of extended-spectrum β-lactamase (ESBL)-producing strains merely adds to the problem, again highlighting the pressing need for novel antibiotics with predictable action against resistant uropathogens [4].

Sulopenem, a new-generation penem antibiotic, offers a potential solution by virtue of its broad-spectrum activity, including against multidrug-resistant Gram-negative bacteria [5]. Unlike other carbapenems, sulopenem is available both intravenously and orally as prodrugs, adding value to its use in the inpatient context [6]. Mechanistically, it differs from previous β-lactams in that it shows high affinity for both serine-based penicillin-binding proteins (PBPs) and L,D-transpeptidases that effectively inhibit both classic and alternative peptidoglycan cross-linking pathways. Its stability against the majority of β-lactamases and favorable pharmacokinetic profile make it a strong contender for uUTIs, particularly where there is high resistance to standard therapy [5]. These characteristics, combined with sulopenem's oral form, make it particularly attractive for outpatient therapy, where an oral step-down regimen can potentially avoid inpatient hospitalization and comply with antimicrobial stewardship programs [5,6]. Despite being promising, there is very limited aggregate clinical evidence regarding the safety and effectiveness of sulopenem for uUTIs, and this warrants a systematic review of extant information [7].

The growing danger of antimicrobial resistance (AMR) has spurred the development of new antibiotics that maintain efficacy with reduced resistance development [8]. Sulopenem's unique structure and mechanism of action-binding to penicillin-binding proteins and interrupting bacterial cell wall synthesis-could be better than earlier β-lactams [6]. For example, sulopenem forms covalent adducts with LdtMab transpeptidases through opening of the β-lactam ring, which varies among enzyme isoforms [9]. This differential inhibition can enhance efficacy against resistant strains [9]. Additionally, its oral bioavailability could reduce hospitalizations via step-down treatment in line with antimicrobial stewardship programs [5]. Nonetheless, its utility for clinical application remains unproven in the absence of robust comparative trials identifying its effectiveness against current first-line agents.

This systematic review aims to consolidate available evidence on sulopenem’s effectiveness, safety, and resistance profile in treating uUTIs. By analyzing data from randomized controlled trials, observational studies, and microbiological surveillance reports, we seek to determine whether sulopenem represents a viable alternative in an era of escalating antibiotic resistance. Furthermore, we explore its potential integration into treatment guidelines and its implications for future antimicrobial development. Given the pressing need for new UTI therapies, this review provides a timely assessment of sulopenem’s place in uUTI management. By synthesizing clinical and microbiological data, we aim to inform clinicians, researchers, and policymakers about its advantages, limitations, and optimal use. The findings may also guide future research directions, particularly in optimizing dosing strategies and evaluating long-term resistance patterns. Ultimately, this work contributes to the broader effort of combating AMR while improving patient outcomes in uUTI treatment.

## Review

Methods

**Figure 1:**
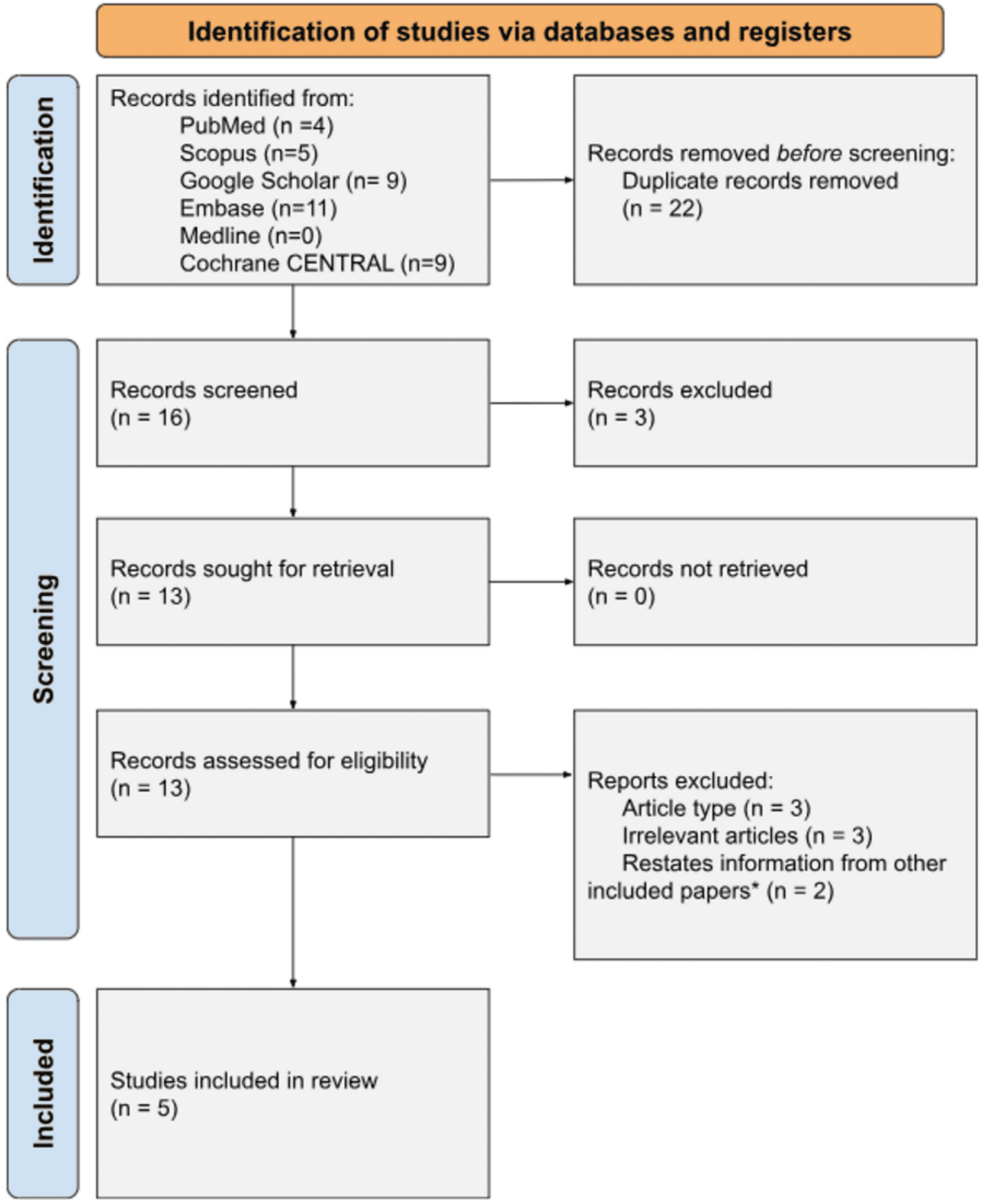
Preferred Reporting Items for Systematic Reviews and Meta-Analyses (PRISMA) flow diagram illustrating the screening and selection process for studies included in the analysis. *We found multiple studies by Dunne to contain the same numerical values and included only the original publication [11].

The methodology followed the 2020 Preferred Reporting Items for Systematic Reviews and Meta-Analyses (PRISMA) recommendations to ensure transparency, reproducibility, and rigorous reporting of the review process [10]. The systematic approach to identifying, selecting, and analyzing studies adhered to these established standards. A flow diagram summarizing the review process is presented in Figure 1. Random effects model was used and the meta-analysis was conducted using RevMan (The Cochrane Collaboration).

We determined important outcome factors such as clinical cure, microbiological eradication, recurrence rate, and adverse events to determine success and failure in the included trials. All qualifying trials were evaluated for therapeutic success or failure using these criteria.

We identified particular factors of relevance from each study prior to obtaining results. These included subgroup stratifications by resistance profiles (e.g., microbiologically modified intent-to-treat - susceptible (MITT-S) vs. microbiologically modified intent-to-treat - resistant (MITT-R) populations), side effects, recurrence rates, microbiological cure, and Test of Cure (TOC) rates. This guaranteed comparability between trials and uniformity in data extraction.

Search Methodology

To evaluate the efficacy and safety of sulopenem in the treatment of uUTIs, we conducted a systematic review of the available literature across multiple scientific databases. These included PubMed, Scopus, Google Scholar, Embase, Medline, and the Cochrane Library. We aimed to identify key clinical studies, randomized controlled trials, and observational studies assessing the role of sulopenem in managing uUTIs.

The search was comprehensive and focused on studies published until March 28, 2025, using an optimized search strategy designed to capture all relevant publications. The strategy employed specific MeSH terms and keywords such as "uncomplicated urinary tract infection", "uncomplicated UTI", "uUTI", and "sulopenem". For a detailed breakdown of the search methodology for each database, refer to Table 1 below.

**Table 1:** MeSH Search Term Strategy

Database	Search String
PubMed	("uncomplicated urinary tract infection"[MeSH] OR "uncomplicated UTI"[TIAB] OR "uUTI"[TIAB]) AND ("sulopenem"[MeSH] OR "sulopenem"[TIAB])
Scopus	TITLE-ABS-KEY ("uncomplicated urinary tract infection" OR "uncomplicated UTI" OR "uUTI") AND TITLE-ABS-KEY ("sulopenem")
Google Scholar	(intitle:"uncomplicated UTI" OR intitle:"uncomplicated urinary tract infection" OR intitle:"uUTI") AND ("sulopenem")
Embase	('uncomplicated uti' OR 'uncomplicated urinary tract infection' OR 'uuti') AND 'sulopenem'
Medline (via Ovid)	("uncomplicated urinary tract infection".mp. OR "uncomplicated UTI".mp. OR "uUTI".mp.) AND ("sulopenem".mp.)
Cochrane Central Register of Controlled Trials (CENTRAL)	("uncomplicated urinary tract infection" OR "uncomplicated UTI" OR "uUTI") AND ("sulopenem")

Eligibility Criteria

Studies were selected based on predefined eligibility criteria encompassing population, intervention, comparison, outcomes, and study design (PICO framework). Inclusion criteria focused on English-language, peer-reviewed journal articles and conference proceedings reporting outcomes of sulopenem in adult patients with uUTIs, with or without comparisons to standard-of-care antibiotics such as fluoroquinolones, β-lactams, or fosfomycin. Exclusion criteria eliminated non-comparative studies, non-primary research (e.g., reviews, editorials), non-English publications, and studies focusing on complicated UTIs or infections caused by multidrug-resistant organisms outside the scope of uUTI. Additional exclusion criteria targeted redundant datasets, studies lacking quantitative outcome data, and analyses without stratification by treatment regimen. The full eligibility criteria are detailed in Table 2.

**Table 2:** Eligibility Criteria for the Inclusion and Exclusion of Studies

Category	Inclusion Criteria	Exclusion Criteria
Population	Adult humans diagnosed with uncomplicated urinary tract infections (uUTIs)	Studies involving pediatric populations, complicated UTIs, or non-human subjects
Intervention	Sulopenem monotherapy or combination therapy for uUTI treatment	Studies not involving sulopenem as a treatment
Comparison	Studies with or without a comparator (e.g., sulopenem vs. standard-of-care antibiotics such as fluoroquinolones, β-lactams, or fosfomycin)	Studies comparing treatments not involving sulopenem
Outcomes	Quantitative results for at least one clinical outcome (e.g., microbiological cure rate, clinical resolution, recurrence rate, safety, adverse effects)	Studies lacking quantitative outcome data
Study Design	Primary research articles including randomized controlled trials (RCTs), cohort studies, case-control studies, and comparative/non-comparative observational studies	Reviews, meta-analyses, health technology assessments, and non-primary research
Language	English-language publications	Publications in languages other than English
Publication Type	Peer-reviewed journal articles and conference proceedings	Abstracts, books, book chapters, grey literature, or unpublished data
Analysis	Studies with analysis stratified by treatment regimen	Studies without stratified analysis by treatment regimen
Patient Population	Unique patient populations with distinct conclusions	Studies with redundant patient populations and similar conclusions

Results

A total of five studies met the predefined inclusion criteria and were included in this comprehensive systematic review. These studies evaluated the efficacy, safety, and pharmacodynamics of sulopenem in the treatment of UTIs, particularly in the context of antibiotic resistance. Study designs ranged from large multicenter randomized controlled trials to advanced pharmacokinetic/pharmacodynamic (PK/PD) modeling studies using in vitro infection models. Table 3 summarizes the key characteristics of the included studies, detailing study design, sample size, recruitment criteria, and data collection methods.

**Table 3:** Characteristics of Included Studies uUTI: uncomplicated urinary tract infection, PK/PD: pharmacokinetic/pharmacodynamic, ESBL: extended-spectrum beta-lactamase, mMITT: microbiologically modified intent-to-treat

Author (Year)	Study Design	Sample Size, N	Recruitment	Collection Summary
Dunne (2020) [11]	Prospective, randomized, double-blind Phase 3 trial	N = 1,671 adult women	Adult women with pyuria, bacteriuria, and uUTI symptoms were enrolled and randomized into two treatment groups.	Data on clinical and microbiologic outcomes were collected at end of treatment and test-of-cure visits.
Henriksen (2021) [12]	Retrospective re-analysis + Prospective trial (SURE-1)	N ≈ not clearly stated for re-analysis; SURE-1 trial (sulopenem vs ciprofloxacin) used published sample	Patient-level data from a 2020 study and participants from the SURE-1 trial were included for comparison.	Data were re-analyzed per 2019 FDA guidance, comparing microbiologic and clinical responses across trials.
VanScoy (2023a) [13]	In vitro, one-compartment, dynamic infection model (PK/PD modeling study).	N = one E. coli isolate (dose-fractionation) + 10 Enterobacterales isolates	Not applicable (lab-based study using bacterial isolates, not human subjects).	Simulated human plasma and urine sulopenem concentration-time profiles were used to expose bacterial isolates over 24 hours (for dose-fractionation) and five days (for dose-ranging); bacterial burden and resistance profiles were quantified on agar plates.
VanScoy (2023b) [14]	Five-day hollow-fiber dynamic in vitro infection model (PK/PD modeling study).	N = four clinical Escherichia coli isolates.	Not applicable; four E. coli isolates (fluoroquinolone-resistant, ESBL+, ST131) were selected for laboratory testing.	Simulated sulopenem 500 mg by mouth every 12 hours urine concentration-time profiles were applied to the isolates over five days in a hollow-fiber model; bacterial burden and resistance were tracked using agar plating at multiple time points.
Puttagunta (2025) [15]	Phase 3 randomized, double-blind, double-dummy, active-controlled trial	N = 2222 randomized; mMITT population = 990; mMITTR = 67	Adult women with uUTI were recruited and randomized to receive either sulopenem/probenecid or amoxicillin/clavulanate.	Clinical and microbiologic outcomes were evaluated at the Test of Cure (TOC) visit, focusing on overall success (clinical + microbiologic) in the mMITT population.

Microbiological and Clinical Efficacy

Sulopenem demonstrated consistent efficacy against uropathogens in uUTIs, with performance varying based on baseline antibiotic susceptibility. According to Dunne et al., in patients with ciprofloxacin-resistant pathogens (MITT-R population), sulopenem achieved significantly higher TOC success rates (62.6%, 92/147) compared to ciprofloxacin (35.0%, 50/139) [11]. However, in ciprofloxacin-susceptible infections (MITT-S), ciprofloxacin remained more effective (78.6%, 326/415) than sulopenem (66.8%, 247/370) [11]. A similar trend was observed in a separate study by Henriksen et al., where sulopenem/probenecid showed a 76.6% microbiological response rate at day 12, slightly lower than ciprofloxacin (79.1%) [12].

When compared to amoxicillin/clavulanate (AMC), sulopenem/probenecid demonstrated non-inferiority in the overall mMITT population and even superiority in the mMITT-S subgroup (61.7%, 296/480 vs. 55.0%, 243/442 for AMC) [12,15]. However, in the mMITT-R subset, AMC showed a numerically higher success rate (68.0%, 17/25) than sulopenem/probenecid (52.4%, 22/42) [12].

Pharmacodynamic Profile and Dose Optimization

Sulopenem’s antibacterial activity was strongly correlated with the time its free drug concentration remained above the minimum inhibitory concentration (MIC) (f%T > MIC). Net bacterial stasis required a median f%T > MIC of 40.9%, while 1-log10 and 2-log10 colony-forming unit (CFU)/mL reductions required 50.2% and 62.6%, respectively [13]. The 500 mg every 12 hours dosing regimen effectively suppressed bacterial growth, reducing initial inoculum levels from 1.0 × 10⁶ CFU/mL to below detectable limits (<1 log10 CFU/mL) while preventing the emergence of resistant subpopulations [14]. This regimen performed comparably to meropenem and outperformed levofloxacin, which failed to show meaningful antimicrobial activity [14].

Pooled Efficacy and Safety Findings

**Figure 2:**
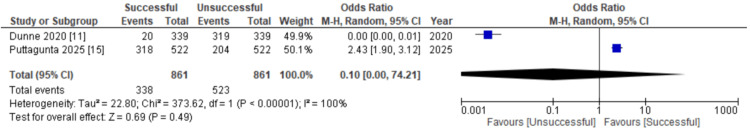
Efficacy of Sulopenem in uUTIs: Pooled Test-of-Cure Analysis. uUTI: uncomplicated urinary tract infection

A meta-analysis incorporating data from Dunne et al. and Puttagunta et al. revealed variability in sulopenem’s TOC success rates, ranging from 5.9% (20/339) to 60.9% (318/522) (Figure 2) [11,15]. This discrepancy may reflect differences in study populations, resistance profiles, or methodological approaches. Despite these variations, sulopenem was well-tolerated, with no reported deaths in clinical trials and a safety profile consistent with other β-lactam antibiotics [11,15].

Nonresponse and Resistance Considerations

A notable proportion of patients did not achieve clinical cure with sulopenem, particularly in the MITT-R group (33.3% nonresponders, 49/147) [11]. This underscores the importance of appropriate patient selection, especially in settings with high fluoroquinolone resistance. Nevertheless, sulopenem’s reliable PK/PD profile and efficacy against resistant pathogens position it as a promising oral option for uUTIs in the era of escalating antimicrobial resistance [13-15].

Risk of Bias

**Figure 3:**
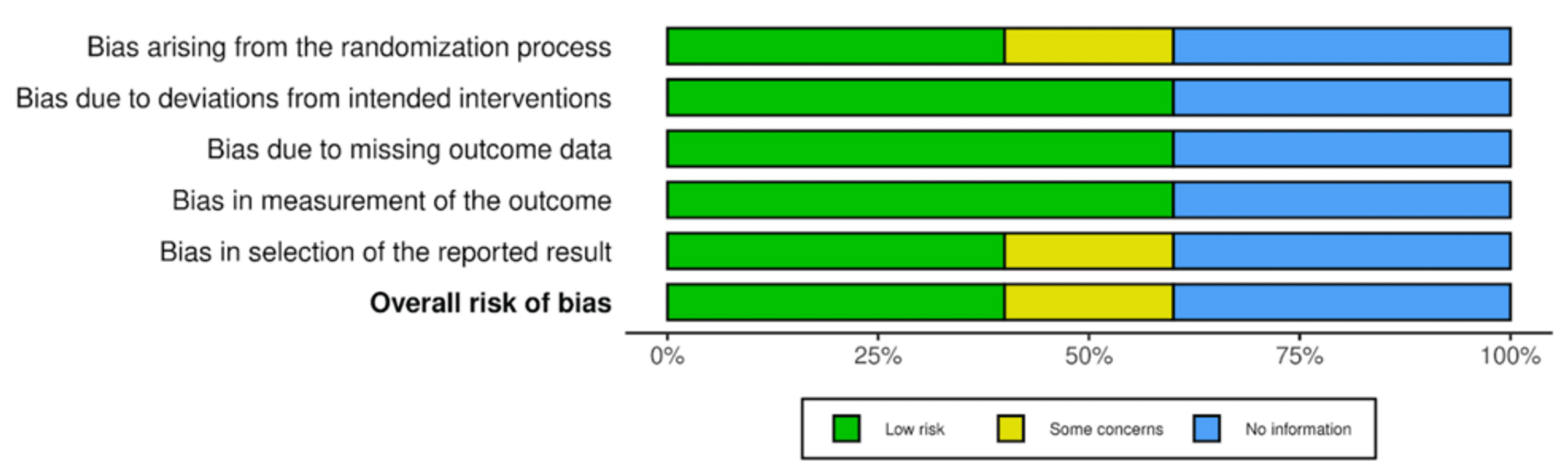
Weighted bar plot showing the distribution of risk-of-bias judgments across bias domains using the RoB 2.0 tool. Each bar represents the proportion of studies categorized as low, some concerns, or high risk of bias within each domain, providing an overview of methodological quality across the included studies. Image created using Risk-of-bias VISualization (robvis) [16].

**Figure 4:**
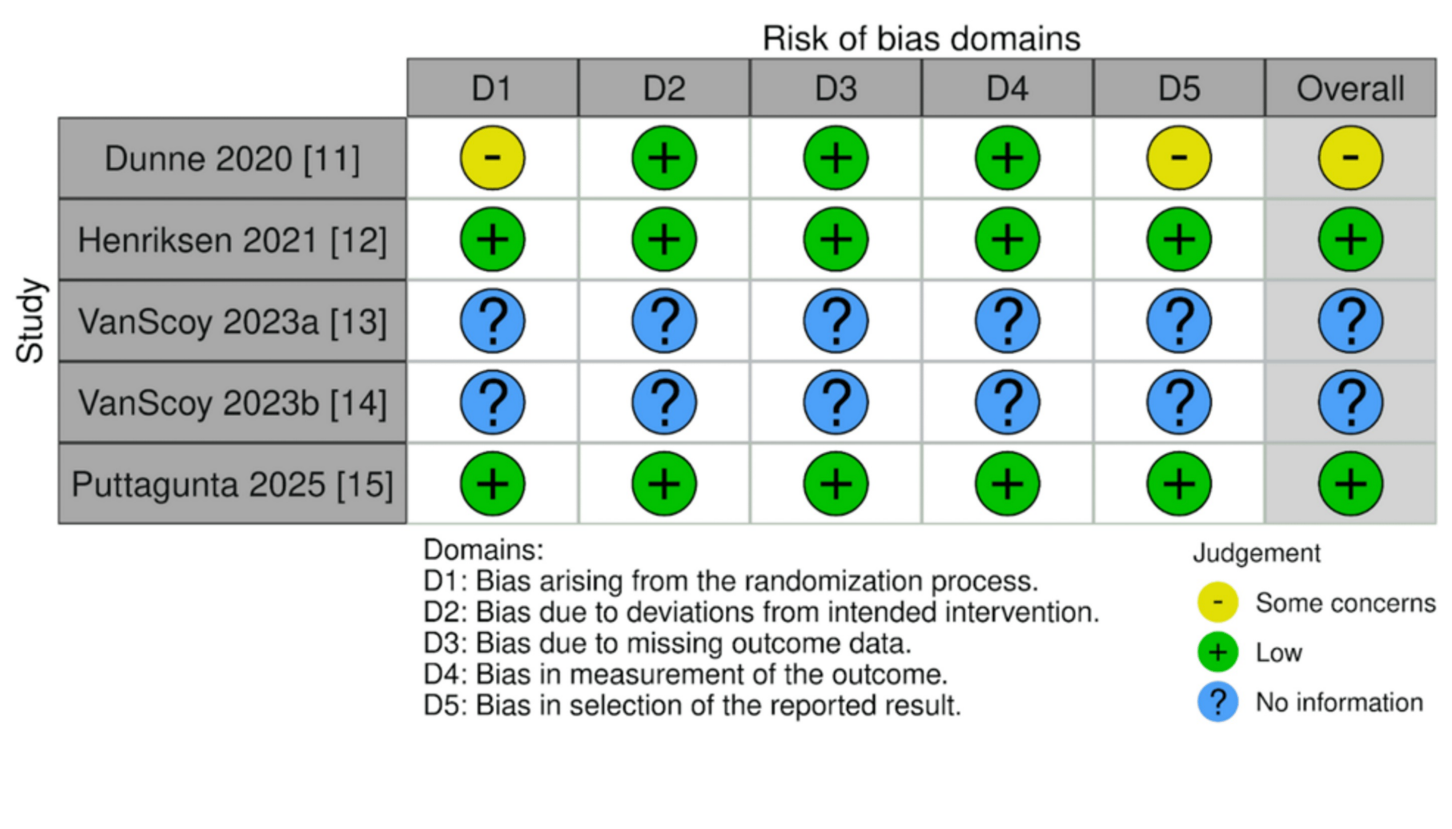
Traffic light plot displaying domain-level risk-of-bias judgments for each included study using the RoB 2.0 tool. Each row represents an individual study, while each column corresponds to a specific bias domain. Image created using Risk-of-bias VISualization (robvis) [16].

The risk of bias assessment for the included studies was conducted using the RoB 2.0 tool, revealing variability in bias levels across different studies (Figure 3) [16]. Notably, Dunne et al. [11] demonstrated some concerns in overall risk of bias, primarily due to issues in D1 (randomization process) and D5 (selection of reported results). In contrast, Henriksen et al. [12] and Puttagunta et al. [15] showed a low risk of bias across all domains. The two in vitro studies by VanScoy et al. [13,14] were evaluated separately due to their non-clinical nature and were not assessed using RoB 2.0 but discussed in terms of internal validity and transparency of reporting (Figure 4).

Summary of Outcomes

The results of the meta-analyses conducted in this study are analyzed using GRADE in Table 4 [17]. The variable accessing the efficacy of sulopenem in treating uUTI analyzed using a meta-analysis was its success rate measured at the Test-of-Cure (Day 12). 

**Table 4:** Grading of Recommendations Assessment, Development and Evaluation (GRADE) Analysis of Meta-Analysis Following GRADEpro Guidelines. CI: confidence interval №: Number ⨁⨁◯◯: Low Explanations a. The two studies included in the meta-analysis presented opposing results. b. Only two studies were found and included in this meta-analysis. From GRADE Handbook [17].

Certainty assessment							№ of patients		Effect		Certainty	Importance
№ of studies	Study design	Risk of bias	Inconsistency	Indirectness	Imprecision	Other considerations	Success rates	Unsuccesful treatment rates	Relative (95% CI)	Absolute (95% CI)		
Efficacy of Sulopenem at Test-of-Cure (Day 12) using Successful vs Unsuccessful Rates (assessed with: Successful vs Unsuccessful Rates)												
2	randomised trials	not serious	serious^a^	not serious	serious^b^	none	338/861 (39.3%)	523/861 (60.7%)	not estimable	not estimable	⨁⨁◯◯ Low^a,b^	CRITICAL

Discussion

The safety and effectiveness of sulopenem against uUTIs were assessed in the present systematic review and meta-analysis that pooled information from five major studies involving pharmacodynamic modeling and randomized controlled trials [11-15]. In resistant uropathogen-infected patients, sulopenem generally equaled or exceeded the activity of various standard-of-care antibiotics, especially in the MITT-R subgroups. While Puttagunta et al. [15] established that sulopenem was not inferior to amoxicillin/clavulanate in the entire mMITT population and even better in the mMITT-S subgroup, Dunne et al. [11] also reported that sulopenem was superior to ciprofloxacin among patients with ciprofloxacin-resistant pathogens. According to these observations, sulopenem also has a lot of therapeutic value as an oral therapy option, particularly in situations where β-lactam or fluoroquinolone resistance is common.

In addition to these positive outcomes, a few limitations need to be noted. Sulopenem had a lower clinical success rate than ciprofloxacin in patients with susceptible pathogens [11,12] and diverging response rates in MITT-R populations between studies [11,15]. Difference in study designs, patient cohorts, mechanisms of resistance in bacteria, or application of recent FDA trial recommendations-which would have led to more demanding definitions of the outcomes-would be the cause of these differences [12]. Our meta-analytic estimate was also less than optimal owing to large amounts of heterogeneity and large confidence intervals in the pooled efficacy data. These contradictions are also represented in our GRADE analysis that gave sulopenem's clinical effectiveness a poor score in certainty of evidence mainly because of imprecision and inconsistency.

Pharmacodynamic studies validated sulopenem's clinical promise by indicating strong in vitro activity against resistant Enterobacterales. During a five-day course of treatment, VanScoy et al. [13,14] demonstrated that oral 500 mg every 12 hours regimen repeatedly resulted in bacterial stasis and interrupted the growth of resistance. These PK/PD results confirm the regimen dosage now being tested in clinical trials and its capacity to sustain therapeutic concentrations above the MIC for an adequate amount of time, an absolute pharmacodynamic determinant of β-lactam antibiotic success [18]. It should be noted that while in vitro models yield useful data, their use in clinical application is impaired since they do not reflect host immune responses, tissue penetration, nor patient variability [19].

Nausea and diarrhea were the predominant side events and sulopenem's safety profile in studies was comparable with other β-lactam drugs [11,15]. Most significantly, no cases of death or serious adverse effects secondary to treatment were noted, confirming its place as an outpatient-safe option [11-15]. In addition to decreasing the requirement for intravenous therapy and inpatient admission, sulopenem's oral route also supports antimicrobial stewardship through decreasing total healthcare costs and the risks of inpatient therapy [20].

Large-scale, head-to-head comparison trials of sulopenem with new oral antibiotics such as fosfomycin, pivmecillinam, and other carbapenem analogs must be the first priority of future studies. For generalizability, these studies must enroll various groups of patients, such as older individuals and recurrent uUTIs patients. Future studies must establish the long-term effect of sulopenem exposure on recurrence rates, emergence of resistance, and disruption of the microbiota. To guide formulary placement and policy recommendations, cost-effectiveness and patient-reported outcome economic research will be critical as well [21]. Surveillance studies and in vitro models will be charged with tracking resistance patterns to the utilization of sulopenem, particularly in regions of high extended-spectrum beta-lactamase (ESBL) prevalence. Lastly, additional clarification of the drug's molecular interaction with various penicillin-binding proteins will reveal possibilities for complementary pairings and guide the design of subsequent β-lactam agents [22].

## Conclusions

Sulopenem has shown encouraging safety and effectiveness for the treatment of simple UTIs, especially in patients with infections caused by bacteria resistant to β-lactams or fluoroquinolones. It bears promise to be a significant addition to the antibacterial arsenal, supported by the best oral bioavailability, broad-spectrum activity, and ability to sustain pharmacodynamic targets. Given the scarcity of long-term data and the heterogeneity in clinical response, sulopenem should be given sparingly-along considerations for patient profiles and patterns in local resistance. However, the evidence is not currently strong as in vitro results do not always translate to clinical effectiveness. Comparative studies coupled with continuous surveillance will need to be conducted to assure its place in contemporary uUTI management and promise in an era of antibiotic resistance. Furthermore, there is a need for region-specific guidelines or surveillance to support real-world adoption.
